# Detection of carbon dioxide embolism by transesophageal echocardiography during transanal/perineal endoscopic surgery: a pilot study

**DOI:** 10.1038/s41598-022-24888-x

**Published:** 2022-11-28

**Authors:** Yoshiko Matsumoto, Suguru Hasegawa, Ryo Ohno, Ryuji Kajitani, Taro Munechika, Hideki Nagano, Akira Komono, Naoya Aisu, Gumpei Yoshimatsu, Yoichiro Yoshida, Kazuya Murayama, Kenji Shigematsu, Kozaburo Akiyoshi

**Affiliations:** 1 Department of Gastroenterological Surgery, Fukuoka University Hospital, 7-45-1 Nanakuma, Jonan-ku, Fukuoka, Japan; 2 Department of Anesthesia, Fukuoka University Hospital, 7-45-1 Nanakuma, Johnan-ku, Fukuoka, Japan

**Keywords:** Gastroenterology, Cardiovascular biology

## Abstract

The transanal/perineal (ta/tp) endoscopic approach has been widely used for anorectal surgery in recent years, but carbon dioxide embolism is a possible lethal complication. The frequency of this complication in this approach is not known. In this study, we investigated the frequency of intraoperative (including occult) carbon dioxide embolism using transesophageal echocardiography. Patients who underwent surgery via the ta/tp approach and consented to participate were included. Intraoperative transesophageal echocardiography was used to observe the right ventricular system in a four-chamber view. Changes in end-tidal carbon dioxide (EtCO_2_), oxygen saturation (SpO_2_), and blood pressure were taken from anesthesia records. Median maximum insufflation pressure during the ta/tp approach was 13.5 (12–18) mmHg. One patient (4.8%) was observed to have a bubble in the right atrium on intraoperative transesophageal echocardiography, with a decrease in EtCO_2_ from 39 to 35 mmHg but no obvious change in SpO_2_ or blood pressure. By lowering the insufflation pressure from 15 to 10 mmHg and controlling bleeding from the veins around the prostate, the gas rapidly disappeared and the operation could be continued. Among all patients, the range of variation in intraoperative EtCO_2_ was 5–22 mmHg, and an intraoperative decrease in EtCO_2_ of > 3 mmHg within 5 min was observed in 19 patients (median 5 mmHg in 1–10 times).Clinicians should be aware of carbon dioxide embolism as a rare but potentially lethal complication of anorectal surgery, especially when using the ta/tp approach.

## Introduction

The transanal/transperineal (ta/tp) endoscopic approach is the only approach from below that can be used in anorectal endoscopic surgery and is useful in cases of a narrow pelvis, bulky tumor, or obesity, where it is difficult to reach the deep pelvis via a transabdominal approach^1–3^. However, with the growing number of cases, there has been an increase in the number of reports of carbon dioxide embolism (CDE) as a complication when using this technique^4–6^. CDE is a potentially fatal complication but is rare in laparoscopic surgery, with a frequency of 0.0014–0.6%^7^. On the other hand, it has been suggested that the incidence of CDE is higher with the ta/tp endoscopic approach (0.39–3.8%) than with laparoscopic surgery^8,9^. However, the exact incidence of CDE when using the ta/tp approach and ways to prevent and respond to it remain unclear.

Clinically significant CDE has a mortality rate as high as 28%^10^. In the management of CDE, early diagnosis and treatment are important for improving prognosis. Transesophageal echocardiography (TEE) is useful for the early diagnosis of CDE and is reported to be able to detect asymptomatic CDE by confirming bubbles in the right heart system. Studies using TEE in laparoscopic surgery have detected intracardiac bubbles in 69% of cholecystectomies, 17.1% of radical prostatectomies, and 100% of hysterectomies^11–13^. However, there are still few relevant reports on CDE using the ta/tp approach. The aims of this study were to investigate the frequency of subclinical CDE detected by monitoring with TEE during anorectal surgery and to find opportunities for early diagnosis of CDE.

## Patients and methods

Patients who underwent surgery via the ta/tp approach for anorectal disease and who consented to participate in the study were included. Patients who were deemed unsuitable for TEE or who did not provide consent were excluded. Surgery was performed using the ta/tp approach as previously reported^3,14–16^. Briefly, total mesorectal excision was performed simultaneously (two-team approach) with the laparoscopic approach in the head-down position. Using the ta/tp approach, CO_2_ was insufflated using an AirSeal system (ConMed, Utica, NY) with high-flow smoke evacuation settings, and surgery was mainly performed using electrocautery. Ultrasonic incision devices and vessel sealers were used for larger vessels. Intraoperative TEE was used to observe the right ventricular system in a four-chamber view at the following time points: (1) before and after induction of anesthesia,(2) immediately after the start of insufflation,(3) immediately after starting the ta/tp approach,(4) when the insufflation pressure of the ta/tp approach was increased,(5) when EtCO_2_ decreased by > 3 mmHg within 5 min,(6) when significant bleeding occurred, and (7) at the end of insufflation. Blood gas tests were also performed at each of these time points. Changes in end-tidal carbon dioxide (EtCO_2_), oxygen saturation (SpO_2_), and blood pressure over time were taken from the anesthesia records. The grade of CDE was assessed by (1) presence of bubbles in the right atrium and (2) changes in EtCO_2_ and vital signs, as reported by Feigl et al. (Table 1)^17^.

**Table 1:** Tübingen Venous Air Embolism Grading Scale.

Grade	Observed changes
0	No air bubbles visible on TEE, no air embolism
1	Air bubbles visible on TEE
2	Air bubbles visible on TEE with decrease of ETCO2 3 mm Hg
3	Air bubbles visible on TEE with decrease of ETCO2 > 3 mm Hg
4	Air bubbles visible on TEE with decrease of ETCO2 > 3 mm Hg and decrease of mean arterial pressure > = 20% or increase of heart rate > = 40% (or both)
5	Same as grade IV causing hemodynamic instability requiring cardiopulmonary resuscitation

*TEE* transesophageal echocardiography; *ETCO2* end-tidal carbon dioxide.

The study was approved by the Fukuoka University Medical Ethics Committee (approval number U19-10-006). Informed consent was obtained from all patients who participated in the study. The study was conducted in accordance with the Declaration of Helsinki and followed the recommendations of the CONSORT Statement.

## Results

Twenty-one patients were enrolled between November 2018 and June 2021. The patient background data are shown in Table 2. The indications for surgery were rectal cancer in 19 cases, chronicgranulomatosis in 1, and local recurrence of cervical cancer in 1. The transanal approach was used in 4 cases and the transperineal approach in 17. The surgical procedures were low anterior resection in 2 cases, intersphincteric resection in 2, abdominoperineal resection in 13, and total pelvic exenteration in 4. The median maximum insufflation pressure when the ta/tp approach was used was 13.5^12–18^ mmHg.

**Table 2:** Background patient characteristics.

Age	Sex	BMI	Indication	c Stage	Comorbidities	Opertion mode
69	M	22.9	Local recurrence of rectal cancer	Recurrence	HT	TPE
77	F	17.6	Rectal cancer	T2N0M0Stage I	None	LAR + LLND
81	M	20.2	Rectal cancer	T4aN3M0cStage III b	Af,BPH	APR + LLND
77	M	25.6	Rectal cancer	T3N2M1a(LN)stage IV	none	APR
79	M	24.6	Rectal cancer	T3N3M1a(Lung)stage IV	DM, Schizophrenia, sarcoidosis	LAR + LLND
39	M	15.3	Chronic granulomatosis		None	APR
59	F	21.7	Rectal cancer	T4b(Vagina)N3 stage III b	None	APR
57	M	21.5	Rectal cancer	T3N3M1b(Liver)stage IV	None	APR + LLND
77	M	22.3	Rectal cancer	T3N0M0 stage II	Epilepsy	APR
91	M	24.8	Rectal cancer	T4aN1M1a(Liver) Stage IV	DM, Angina, AS, HT, Prostate cancer	APR
47	F	15.7	Anal fistula cancer	T4b(Vagina)N3M0 Stage III b	Crohn's disease	TPE
69	M	19.8	Rectal cancer	T4b(Bl, SI, Rt ureter)N3M0 StageIIIb	None	TPE
54	F	35.1	Rectal cancer	T4b(LA, Vagina)N1bM0 StageIIIc	DM, schizophrenia, sarcoidosis	APR
76	M	22	Rectal cancer	T4b(LA)N0M0 Stage2 II	Angina, HT, Af	APR
49	M	24.2	Rectal cancer	T2N0M0 stage I	None	APR
59	F	22	Rectal cancer	T2N2aM0 stage III b	HT	ISR + LLND
80	M	27.9	Rectal cancer	T4b(LA)N1bM0 stage IIIc	Old myocardial infarction	APR
42	F	18.1	Local recurrence of cervical cancer	Recurrence	Graves' disease	TPE
65	M	27.9	Rectal cancer	T2N0M0 stage I	HT	ISR
61	M	19.7	Anal fistula cancer	T3N0M0 stage II	Angina, HT, Crohn's disease	APR
58	F	23.6	Rectal cancer	T3N3M0 stage IIIb	Deep vein thrombosis	APR + LLND

*DM* Diabetes mellitus; *HT* Hypertension; *Af* Atrial fibrillation; *AS* Aortic valve stenosis; *BPH* Benign prostatic hypertrophy; *LA* levator ani muscle; *Bl*: Bladder; *SI* small intestine; *LN* lymph node; *LLND* Latelal lymph node dissection.

**Figure 1:**
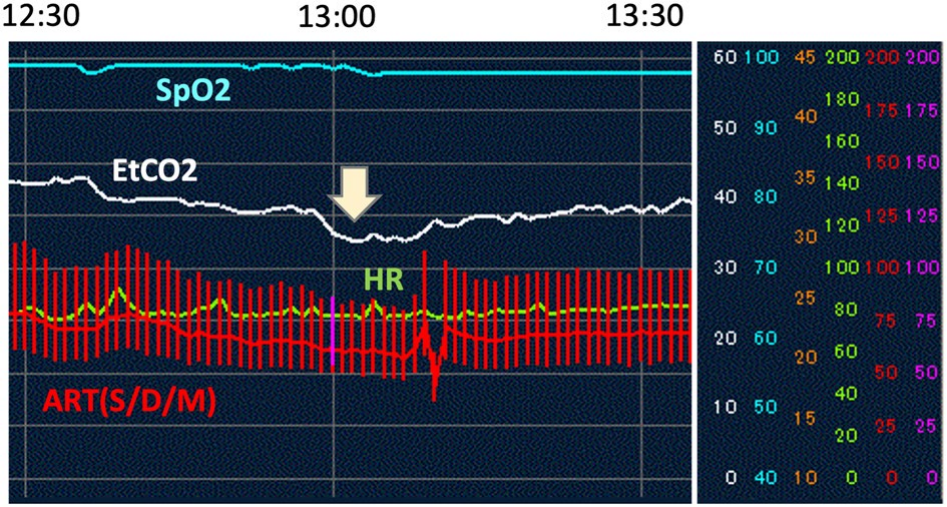
Intraoperative hemodynamic monitoring; TEE was performed immediately after a decrease in EtCO2 (arrow).

The results of Intraoperative TEE monitoring are shown in Table 3. One case (4.8%) was observed to have a bubble in the right atrium; in this case, there was no change in blood pressure, SpO_2_, or pulse rate but there was a decrease in EtCO_2_ from 39 to 35 mmHg, and the patient was classified as grade 3. When the anesthesiologist confirmed the bubble by TEE (Supplemental Movie), the insufflation pressure was decreased from 15 to 12 mmHg to 10 mmHg until the surgeon identified the bleeding site, which was found to be a branch of the internal pudendal vein. Immediately after the bleeding was stopped, TEE confirmed that the bubble had disappeared. EtCO2 returned to its original value in 15 min and remained stable thereafter (Fig.1). There were no findings of right heart loading, even in the case where a bubble was observed in the right ventricular system by TEE. The surgery was continued using the two-team approach with TEE monitoring and was completed with no further complications.

**Table 3:** Operative parameters.

Operative time (min)	Blood loss (mL)	TEE observations (times)	Max insufflation pressure (mmHg)	Insufflation pressure (Ta/Tp) (range: mmHg)	Intraopeative range of Et-CO_2_ (mmHg)	No. of times EtCO_2_ decreased (> 3 mmHg w/in 5 min)	Dtection of gas bubbles by TEE	Grade of gas embolism*
955	485	14	15	10 ~ 15	32–50	4	Yes	3
348	4	10	13	10 ~ 13	35–42	1	None	0
449	95	13	15	10 ~ 15	35–45	5	None	0
431	46	19	14	10 ~ 14	32–50	3	None	0
696	350	11	18	12 ~ 18	36–45	3	None	0
494	151	17	15	10〜15	34–40	0	None	0
758	98	6	15	12 ~ 15	25–45	4	None	0
863	287	13	17	12 ~ 17	30–40	4	None	0
389	40	12	15	10 〜 15	27–40	3	None	0
286	60	6	14	12 〜 14	32–42	4	None	0
811	757	6	12	12	30–40	4	None	0
941	658	8	12	12	32–43	10	None	0
561	186	6	12	12	30–52	9	None	0
395	43	7	12	12	32–42	1	None	0
303	92	7	15	10 〜 15	28–45	4	None	0
582	55	5	12	12	32–44	5	None	0
351	63	5	15	10 〜 15	30–38	1	None	0
887	497	7	12	12	30–50	4	None	0
455	10	8	15	12 〜 15	35–40	0	None	0
312	5	7	12	12	32–40	3	None	0
468	70	6	12	10 〜 12	30–45	5	None	0

*Tübingen Venous Air Embolism Grading Scale (Table 1).

No bubble was found in the right atrium on TEE in any other cases. Among all patients, the range of variation in intraoperative EtCO_2_ was 5–22 mmHg (median 16.5). A decrease in intraoperative EtCO_2_ by > 3 mmHg within 5 min was observed in 90.5% (19/21) of patients (median 5 mmHg in 1–10 times) but TEE revealed a bubble in the atrium in only 1 case.

## Discussion

The incidence of CDE when performing anorectal surgery via the ta/tp approach has been increasingly reported in recent years^8^. At our institution, symptomatic CDE has occurred in 2 (1.6%) of 120 cases, which is similar to the 3 (4%) of 80 cases reported by Harnsberger et al^9^. On the other hand, occult (asymptomatic) CDE was observed in 1 (4.8%) of our 21 cases in this study, which was lower than the rate at which TEE detected asymptomatic CDE in various organs^11–13^.

The incidence of CDE is reported to be higher with the ta/tp approach than with routine laparoscopic surgery. Possible reasons for this are as follows: (1) ta/tp requires a high insufflation pressure to secure the operative field^18^; (2) the veins around the perineum are relatively large and flow into the systemic circulation; and 3) the high pelvic position results in low central venous pressure, which promotes uptake of CO_2_ easily when a small hole is formed.

A sudden decrease in SpO_2_ or blood pressure accompanied by a rapid decrease in EtCO_2_ should raise suspicion for CO_2_ embolism, which is diagnosed using TEE or transthoracic echocardiography^19,20^. However, the decrease in EtCO_2_ can fluctuate depending on various factors, such as ventilator settings. In our study, a decrease in EtCO_2_ by > 3 mmHg within 5 min, which is an indicator used to grade CDE, was observed a total of 77 times in 19 cases (1–10 times) without any sign of bubbles on TEE. Therefore, comprehensive judgment is necessary rather than relying on only changes in EtCO_2_ as an indicator of CDE. When performing operations in areas of the perineum with a well-developed venous plexus, it is important to be vigilant for CDE and changes in EtCO_2_, SpO_2_, or vital signs. A visible venous lumen in the absence of bleeding means that the insufflation pressure is higher than the central venous pressure, which increases the risk of CDE. If respiratory and circulatory changes are detected, CDE should be suspected and TEE used for a definitive diagnosis while promptly discontinuing delivery of air.

We have previously had a case of CDE during tp-TPE in which the patient's condition worsened due to delayed diagnosis of CDE and conversion to laparotomy was required^21^. Early detection and management of CDE is important to prevent a deteriorating situation. Early diagnosis of CDE and lowering of the insufflation pressure in the patient with asymptomatic CDE in our present study resulted in prompt disappearance of the bubble without serious complications, and surgery could be continued more safely with monitoring by TEE.

This study has several limitations. First, it included only 21 cases. Second, TEE was not performed continuously, so the possibility that some bubbles were missed cannot be excluded. Lack of continuous monitoring might be the reason for wide variation in the detection rate of bubbles across studies (17.1–100%)^11–13^. Finally, although TEE monitoring may contribute to early detection of CDE, it is not realistic to use this method routinely in the clinical setting because of the cost, equipment, and personnel required, and the need for prolonged placement in the esophagus. However, our experience is that if CDE is suspected, it can be diagnosed immediately by TEE, and if vital signs are stable after insufflation is stopped, surgery can be continued more safely with ta/tp by monitoring with TEE. Both surgeons and anesthesiologists should keep CDE in mind and collaborate closely to detect and manage this complication^19,20^.

## Conclusions

In this study, TEE monitoring in patients undergoing anorectal surgery via a ta/tp approach detected asymptomatic CDE in 1 of 21 cases (4.8%). Clinicians should be aware of this rare but potentially lethal complication in view of the ever-increasing popularity of the ta/tp approach.

## Supplementary Information

Supplementary Video 1.

## Data Availability

The datasets used and/or analyzed in this study are available from the corresponding author upon reasonable request.
